# Using “warm handoffs” to link hospitalized smokers with tobacco treatment after discharge: study protocol of a randomized controlled trial

**DOI:** 10.1186/1745-6215-13-127

**Published:** 2012-08-01

**Authors:** Kimber P Richter, Babalola Faseru, Laura M Mussulman, Edward F Ellerbeck, Theresa I Shireman, Jamie J Hunt, Beatriz H Carlini, Kristopher J Preacher, Candace L Ayars, David J Cook

**Affiliations:** 1 University of Kansas Medical Center, 3901 Rainbow Boulevard, Kansas City, KS, USA; 2 Department of Preventive Medicine and Public Health, University of Kansas Cancer Center, 3901 Rainbow Boulevard, Kansas City, KS, 66160, USA; 3 Alcohol and Drug Abuse Institute, University of Washington, Seattle, USA; 4 Alere Wellbeing (Formerly Free & Clear), Seattle, USA; 5 Department of Psychology & Human Development, Vanderbilt University, Nashville, TN, USA; 6 Formerly of Kansas Department of Health and Environment, Topeka, KS, USA

**Keywords:** Tobacco use disorder, Smoking cessation, Hospital, Randomized clinical trial

## Abstract

**Background:**

Post-discharge support is a key component of effective treatment for hospitalized smokers, but few hospitals provide it. Many hospitals and care settings fax-refer smokers to quitlines for follow-up; however, less than half of fax-referred smokers are successfully contacted and enrolled in quitline services. “Warm handoff” is a novel approach to care transitions in which health care providers directly link patients with substance abuse problems with specialists, using face-to-face or phone transfer. Warm handoff achieves very high rates of treatment enrollment for these vulnerable groups.

**Methods:**

The aim of this study—“EQUIP” (Enhancing Quitline Utilization among In-Patients)—is to determine the effectiveness, and cost-effectiveness, of warm handoff versus fax referral for linking hospitalized smokers with tobacco quitlines. This study employs a two-arm, individually randomized design. It is set in two large Kansas hospitals that have dedicated tobacco treatment interventionists on staff. At each site, smokers who wish to remain abstinent after discharge will be randomly assigned to groups. For patients in the fax group, staff will provide standard in-hospital intervention and will fax-refer patients to the state tobacco quitline for counseling post-discharge. For patients in the warm handoff group, staff will provide brief in-hospital intervention and immediate warm handoff: staff will call the state quitline, notify them that a warm handoff inpatient from Kansas is on the line, then transfer the call to the patients’ mobile or bedside hospital phone for quitline enrollment and an initial counseling session. Following the quitline session, hospital staff provides a brief check-back visit. Outcome measures will be assessed at 1, 6, and 12 months post enrollment. Costs are measured to support cost-effectiveness analyses. We hypothesize that warm handoff, compared to fax referral, will improve care transitions for tobacco treatment, enroll more participants in quitline services, and lead to higher quit rates. We also hypothesize that warm handoff will be more cost-effective from a societal perspective.

**Discussion:**

If successful, this project offers a low-cost solution for more efficiently linking millions of hospitalized smokers with effective outpatient treatment—smokers that might otherwise be lost in the transition to outpatient care.

**Trial registration:**

Clinical Trials Registration NCT01305928

## Background

Post-discharge support is a key component of effective treatment for hospitalized smokers, but very few hospitals provide it [1]. Linking hospitalized smokers with tobacco quitlines is an ideal way to provide supportive contact at discharge. Proactive tobacco quitlines are effective and cost-effective for smoking cessation [2,3]; they are freely available to many US smokers [4]; services are delivered via telephone, which minimizes many access barriers; hospitals do not have to bear the costs of the services; and many quitlines are undersubscribed and eager to increase their reach [5,6].

In the last 10 years, several health care facilities have adopted the practice of providing fax referrals to state quitlines in an effort to link smokers with ongoing support [7-9]. This practice has produced mixed results. Studies conducted within primary care settings document conversion rates from fax referral to enrollment varying from 16-42% [10,11]. Unfortunately, no such studies have been conducted primarily among hospital patients, so the conversion rate from fax referral to enrollment among hospitalized patients is not known. Our own 6-month follow-up data among University of Kansas Hospital (KUMed) patients found that only 5% of patients fax-referred to the quitline reported they had participated in quitline services [12]. Moreover, no studies have yet reported the effectiveness of fax referral on long-term quit rates. Hence, fax referral is a promising but unproven method for linking smokers with post-medical care support.

Integrated Behavioral Health (IBH) and Screening, Brief Intervention, and Referral to Treatment (SBIRT) programs offer another model of linking hospital patients with quitlines post-discharge. IBH is a national initiative to integrate behavioral health services into primary care settings [13,14], and SBIRT is a federal demonstration program to institute a system of universal screening, brief intervention, and referral to treatment for persons with substance abuse in medical care settings [15]. Program evaluation data on 459,599 patients from six states suggest that SBIRT is feasible to implement and results in self-reported improvement in drug and heavy alcohol use, as well as health status, among recipients of services [16]. Some SBIRT services have adopted the practice of “warm handoff.” In this model, health care providers walk the patient to a behavioral health care provider who is co-located in the medical setting. The behavioral health provider enrolls and initiates treatment before the patient leaves the acute-care setting. At present, few published reports of the effectiveness of warm handoff are available. One report found 80% of patients attended their first appointment with their health care provider after being transferred via warm handoff, as opposed to 20% who were referred by word or paper recommendation; another reported warm handoff achieved 80%-90% enrollment, as opposed to 10% enrollment via less intensive referral methods [17,18].

An evaluation of warm handoff as a method of transitioning patients to post-discharge care would be valuable for hospitals that wish to comply with proposed new US Joint Commission Accreditation of Healthcare Organization (Joint Commission) guidelines. These guidelines recommend universal screening for tobacco, brief intervention for all who screen positive, referral for post-discharge treatment, and post-discharge follow-up to assess outcomes and ensure the patient has enrolled in treatment. These measures will encourage hospitals to ensure smokers receive evidence-based treatment after discharge.

This project, Enhancing Quitline Utilization among In-Patients (EQUIP), is funded by the National Heart, Lung, and Blood Institute (U01 HL105232-01) and is part of an NIH Cooperative Agreement. The research teams funded under this cooperative agreement have become a consortium and adopted the name of “CHART” (Consortium of Hospitals Advancing Research on Tobacco). The overall goal of the project described in this article is to determine the relative effectiveness, and cost-effectiveness, of warm handoff versus fax referral in linking hospitalized smokers with quitline services. We hypothesize that, compared to patients receiving fax referral, more patients in the warm handoff condition will enroll in quitline services, have an initial counseling session, and participate in follow-up sessions post discharge. We believe the active ingredient in the intervention is the chance to sample quitline participation prior to hospital discharge and that this experience will lead more smokers to accept follow-up calls after going home. Ultimately, enrolling in quitline services and adhering to counseling should lead to higher rates of cessation. The intervention was designed to be simple, translatable, and sustainable to enhance its potential for widespread adoption and ultimate impact on public health. This paper describes the study protocol.

## Methods/Design

### Overview of design and setting (Figure 1)

We employ a control-group design with individual randomization to study arms—warm handoff versus fax referral. The study will be conducted over a 4-year period. Participants will be hospitalized smokers from two large hospitals that have dedicated tobacco treatment services. Dedicated hospital treatment staff will screen smokers referred to services for eligibility, and collect informed consent and baseline data. Afterwards, they will randomize patients to study arms, conduct in-hospital intervention, and refer enrolled smokers to quitline according to their study arm. Research staff will collect 1-, 6-, and 12-month follow-up data. Outcome measures and analyses include 30-day point prevalence abstinence at 6 months, biochemically verified 7-day point prevalence, quitline enrollment and adherence, and cost effectiveness. The tobacco quitline vendor for Kansas will provide process data on counseling enrollment and adherence. We estimate 994 participants will be required to detect the treatment effect. We will recruit the majority of patients (700) from the University of Kansas Hospital (KUMed) in Kansas City, and the remaining 294 from Stormont Vail Regional Health Center in Topeka, Kansas. KUMed is a 600-bed academic medical center; its tobacco treatment service (UKanQuit) was founded in 2006. Stormont Vail is a 586-bed acute care referral center for Northeast Kansas; its tobacco treatment service is new and was developed for the purpose of implementing this trial. The training and experience of UKanQuit staff range from Associates Degrees with lengthy experience in substance abuse treatment to Masters’ Degrees in counseling. Staff has received tobacco treatment specialist trainings at accredited programs.

**Figure 1:**
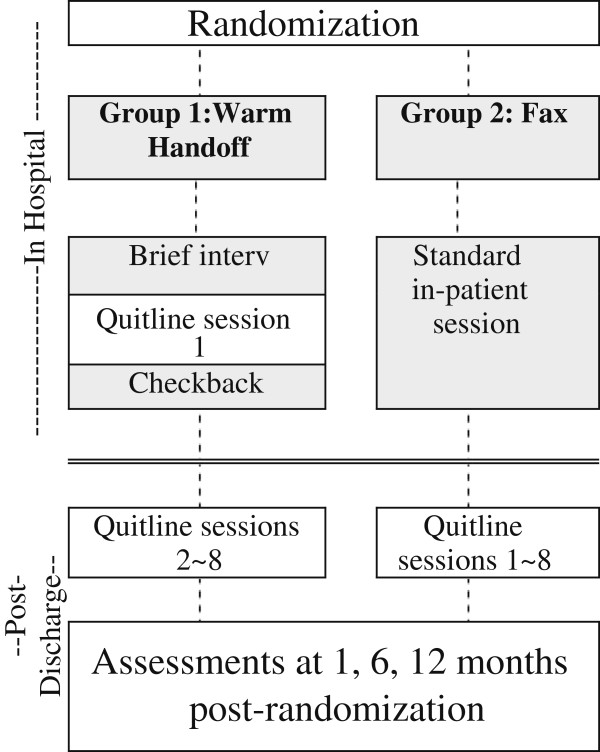
Overview and study design of EQUIP – a randomized controlled trial.

### Identifying hospitalized smokers

Approximately 800 patients are referred to UKanQuit at KUMed each year. Patients are referred via provider orders and self-referral. Numerous providers can enter orders for tobacco treatment at any time during a patients’ hospital stay. Self-referral is via automated prompts delivered to patients by the electronic medical record (EMR) during admission. As nurses record information from patients admitted to their units, the EMR requires them to identify the smoking status of all patients and to ask all smokers if they would like to talk to a tobacco treatment specialist during their hospital stay. The patients who respond “yes” and also those whose physicians request treatment orders are placed on an electronic list for the tobacco treatment service. In addition to referrals, the UKanQuit service staff can view and actively recruit from a list of all smokers in the hospital at any given time. Stormont Vail has no tobacco treatment order or request system. Their smokers will be identified via a tobacco user list generated from the EMR.

### Recruitment, baseline assessment, consent, and randomization (Figure 2)

At both study sites, UKanQuit staff will receive training regarding screening participants, collecting consent, and conducting the warm handoff intervention. UKanQuit staff already provides fax referral to quitline as a part of their usual duties. During the study recruitment period, UKanQuit staff will, as usual, visit all referred hospitalized smokers at bedside. Patients may enroll in the study only once. Study staff will first check the patients’ medical record against a list of medical records for all current study participants to prevent enrolling the same patient multiple times. Staff will briefly describe the study and screen for eligibility/interest in participating in the study. They will provide treatment as usual, including fax referral to quitline, to patients who are not eligible or willing to participate. Among patients who are willing to participate, staff will collect informed consent via a simplified, one-page consent form developed for the trial. UKanQuit staff will then conduct a brief baseline assessment, conduct random assignment, and provide intervention/referral according to the study arm to which the patient was assigned. In order to ensure that equal numbers of patients are assigned to intervention and control conditions, we will conduct block randomization between the two sites and across the three sources of referral (proactive recruitment among all smokers, physician orders, and patient requests to see specialists).

**Figure 2:**
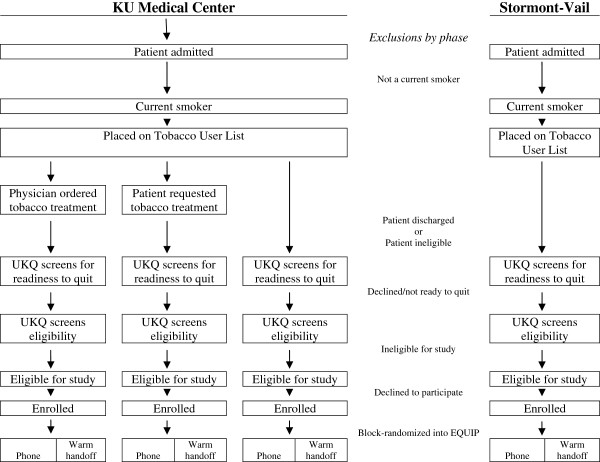
Recruitment of participants into EQUIP.

### Participants

Hospital patients who smoke and have been referred for tobacco treatment will be eligible for enrollment. Eligibility criteria are listed in Table 1. We exclude non-Kansans because residents of other states may receive quitline services from a different vendor, which would confound intervention effects and make it difficult to obtain quitline adherence data. Participants who are ineligible or who refuse to participate during one hospitalization will be screened and enrolled at a later date if they meet study criteria and agree to participate during subsequent hospitalizations.

**Table 1:** Eligibility criteria


·	Current smoker (within the past 30 days)
·	Aged 18 years or older
·	Lives in Kansas
·	Wishes to remain abstinent from tobacco post-discharge
·	Plans to remain a resident of Kansas post-discharge
·	Speaks English or Spanish
·	No significant co-morbidity or issue that would prevent participation
	(e.g., acute life-threatening medical illness, communication barriers,
	altered mental status, etc.) as determined by hospital or research staff
·	Has access to a telephone post-discharge
·	Does not have another household member participating in this study
·	Is not currently pregnant

### Intervention and control arm procedures (Table 2)

**Table 2:** In-patient treatment, warm handoff versus fax

**Warm Hand-Off**	**Fax**
*Staff brief intervention and warm handoff (5 min):*	*Staff standard in-patient session: (20 min):*
· Assess withdrawal, need for medication change	· Assess withdrawal, need for medication change
· Describe warm handoff process	· Conduct assessment of smoking history, interest in quitting
· Provide cessation brochure	· Explore thoughts/feelings toward quitting
· Perform call, leave room	· Provide cessation brochure
· Notify patients’ nurse that patient is talking to quitline	· Provide medication education
*Quitline session (20 min):*	· Build plan to stay quit
· Collect minimum data set	· Describe fax referral process
· Explore thoughts/feelings toward quitting	· Ask if patient requests cessation medication script on discharge
· Provide medication education	
· Build plan to stay quit	
· Schedule next call	
*Staff check-back (5 min):*	
· Ask patient how session went	
· Ask if patient requests cessation medication script on discharge	

Patients in both study arms will receive the hospitals’ standard cessation brochure with information and resources on quitting smoking. For patients in the fax-referral group, UKanQuit staff will provide the standard in-hospital intervention and will fax-refer patients to the state tobacco quitline for counseling post-discharge. For patients in the warm handoff group, the UKanQuit staff will provide an abbreviated in-hospital intervention, warm handoff to the quitline, and a brief check-back visit.

#### Fax referral group

UKanQuit, and the tobacco treatment service as Stormont Vail, deliver in-hospital treatment that was adapted from the inpatient intervention provided at Massachusetts General Hospital. UKanQuit screening and intervention procedures are described more fully elsewhere [12,19]. Sessions include (1) assessing withdrawal; (2) working with nurses and physicians to adjust nicotine replacement to keep the patient comfortable while in the hospital; (3) assessing patients’ interest in quitting smoking (e.g., remaining abstinent after discharge); (4) providing a brief motivational intervention to patients not interested in quitting; (5) providing assistance in quitting (arranging for medication scripts on discharge, arranging for follow-up with quitline; developing a quit plan); and (6) documenting treatment in the electronic medical record. UKanQuit intervention procedures are highly standardized and include data collection forms, a quit plan, and intervention checklists to facilitate supervision of counseling procedures by the clinic director and coordinator. Staff fax-refer patients to the quitline on the day they are discharged from the hospital, to ensure patients receive a call from the quitline soon after arriving at their home or residential facility.

#### Warm handoff group

Brief intervention, warm handoff, quitline enrollment/counseling, and check-back are designed to last a total of 30 min. During their initial brief intervention, UKanQuit staff will assess withdrawal, describe warm transfer procedures, perform the handoff, and leave the room. UKanQuit staff will perform the handoff by calling the quitline, notifying the quitline that an inpatient from Kansas is on the line, then transferring the call to the patients’ mobile or bedside hospital phone for quitline enrollment and, time permitting, an initial counseling session with an Alere Quit Coach. During the quitline call, the hospital counselor will notify the patient’s nurse that the patient is participating in quitline telephone counseling. The counselor may use this time to work with the patient’s health care team to adjust inpatient nicotine replacement, if the patient reported withdrawal and craving. After the quitline session, the counselor will check back with the patient and provide further assistance, such as arranging for medication scripts on discharge in consultation with the patients’ health care team. The counselor will document the intervention in the medical record.

#### Troubleshooting handoffs and referrals

As with standard inpatient counseling, UKanQuit staff may initially find patients occupied with hospital procedures, but will return when patients are free to participate in counseling. Similarly, at times counseling sessions are interrupted by hospital procedures. If the procedure will be brief, the counselors will wait and resume the session afterwards. However, if the procedure will be lengthy, the counselor will return at a later time. Should patients be interrupted during a quitline call, the Quit Coach and patient may opt to complete enrollment, which requires approximately 5 min, and schedule the initial counseling session for a later time in the day or for a day post-discharge. Hospital staff carry smart phones to receive pages from hospital units, check emails, and call physicians to discuss patient medications; this will enable them to check with nurses on patients’ availability and perform warm handoffs while present in patients’ rooms.

Arranging for medications at discharge is a very important function of hospital treatment. We instituted the check back so that warm hand off patients would have the opportunity to request changes in inpatient cessation medication as well as medication on discharge, based on their discussions with their quitline coaches.

In the fax group, the patient gets counseling that is similar to quitline counseling, but is provided by the hospital tobacco treatment (UKanQuit) staff. At the close of the session, UKanQuit staff asks the patients if they would like to change their inpatient medication or request a prescription for medication on discharge.

Hence, the fax referral group doesn’t need a check back because the counselor never leaves the room. The check back for warm hand off patients provides an interface between the quitline coach and the hospital staff, and gives equal opportunity for patients in the warm hand off group to obtain cessation medications. If warm hand off to quitlines should become disseminated widely, the check back would be a key role for nursing staff or other care providers, because without it patients could leave the hospital without being offered prescriptions for cessation medications, which would fail to fulfill new Joint Commission guidelines for tobacco treatment.

#### Quitline services

The Kansas Department of Health and Environment (KDHE) contracts with Alere Wellbeing (Formerly Free & Clear) for quitline services. Participants who accept referral to the quitline and enroll in quitline services will receive three to eight proactive counseling calls, depending on their intake assessment and interest in continued counseling. Each call is designed to provide practical expert support to help participants develop problem-solving and coping skills, secure social support, and design a plan for successful cessation and long-term abstinence.

### Hospital tobacco treatment staff fidelity monitoring

We will assess UKanQuit staff fidelity to treatment to ensure both study arm interventions are well implemented and the trial is a valid test of warm handoff versus fax referral. Components of the intervention that require fidelity control include (1) completing all steps of the in-hospital intensive intervention, (2) accurately describing the referral procedure to the patient, (3) making the referral, (4) completing the referral in the correct time frame, and (5) correctly filling out referral forms. Fidelity to components 1–3 will be assessed in person during hospital consults. Fidelity to usual care will be measured using a checklist that outlines intervention components for usual care. Any deviations from usual care, including enhanced care not included in the protocol, will be noted, and hospital staff will receive corrective feedback on this. Research staff will accompany each hospital staff member on a 5% sample of randomly selected hospital sessions and use checklists to assess fidelity to in-hospital study components. Fidelity to components 1 and 4–5 will be assessed for the same sample of visits by records inspection. Research staff will check EMR entries for patients to assess completeness of documentation. Staff will interface with our quitline collaborators to collect dates of referral and copies of referral forms (for fax referrals only) to assess whether referrals were made, and made correctly. Data on fidelity to procedures for each study arm will be entered into a database and reported back to hospital staff on a monthly basis to encourage adherence to protocols.

### Data collection

Baseline assessment will be conducted prior to randomization by UKanQuit staff. Follow-up assessments will be conducted by research assistants; assessments occur at the following post-randomization times and reimbursement levels: 1 month ($20); 6 months ($50), and 12 months (EMR data download for health care utilization only—no reimbursement provided). In addition, participants who provide salivary cotinine samples following the 6-month assessment are reimbursed $100. Research assistants will be fluent in English and Spanish. We will reimburse participants for all assessments. The project has collaborated with other CHART members on the development and use of standardized measures, methods, and data management.

### Study measures (see Table 3)

**Table 3:** Study measures by assessment time point*

**Time points:**	**Baseline**	**Mo. 1**	**Mo. 6**	**Mo. 12**
**Self-reported smoking abstinence**				
30-day point prevalence		✓	✓	✓
7-day point prevalence		✓	✓	✓
Salivary cotinine			✓	
Prolonged abstinence		✓	✓	✓
Time to relapse		✓	✓	✓
Quit date	✓			
**Smoking and quitting history**				
Cigarette use in past 30 days	✓			
No. days smoked in past 30 days	✓			
Age started using tobacco	✓			
No. of quit attempts		✓	✓	✓
No. of cigarettes smoked per day	✓	✓	✓	✓
Medications used to quit		✓	✓	✓
Resources used to quit		✓	✓	✓
Other forms of tobacco, no. of days used	✓	✓	✓	✓
E-cigarette use		✓	✓	✓
Smoking during hospitalization		✓		
Other tobacco during hospitalization		✓		
Time to first cigarette	✓			
Around tobacco users at home/work	✓			
Home smoking restrictions	✓			
Other household smokers	✓			
Stages of change	✓			
Readiness to quit	✓			
**Quitline use**				
Enrolled in quitline			✓	
No. quitline calls completed			✓	
Length of quitline calls			✓	
**Co-morbidities**				
Body mass index	✓			
Co-morbidities	✓			
**Hospitalization factors**				
Health insurance	✓			
Prescription drug coverage	✓			
Length of stay (admit/discharge time/date)	✓			
Discharge diagnoses (primary/secondary)	✓			
Procedure codes, diagnosis-related group (DRG)	✓			
Discharge plan	✓			
Admit via emergency, admitting hospital service	✓			
Transitions in tobacco care		✓		
Hospitalization/access to care**	✓	✓	✓	✓
**Depression and alcohol use**				
Patient Health Questionnaire (PHQ)-2	✓	✓	✓	✓
AUDIT-C***	✓			
**Demographics**				
Race, ethnicity, age, sex, education	✓			
Marital status, income, employment	✓			

*Excluding cost-effectiveness measures.

**From the National Health And Nutrition Examination Survey (N-HANES).

*** Alcohol Use Disorders Identification Test, version C.

We will collect all CHART core measures (see Riley et al., lead paper in this special issue). Our baseline measure is designed to be brief to preserve time for intervention, and minimize the burden on patients and the potential for UKanQuit staff to be interrupted by other hospital staff who must attend to patients. To assess mediation, at 1 month post-randomization we will collect items adapted from the Care Transition Measure (CTM-15) [20], adapted for tobacco treatment, to assess patients’ perceptions of how well the two conditions (warm handoff versus fax) facilitated the transition to outpatient tobacco care. Quitline enrollment is defined as having completed the quitline’s enrollment assessment. Adherence to counseling will be measured via a count variable that can range from 0–5, which corresponds to the number of sessions completed. In addition, we will form a composite measure of completion of guideline-recommended levels of counseling. This will be a binary measure of whether participants completed at least 1 month of counseling follow-up (at least three counseling sessions post-discharge). The data for these measures will be derived from Alere reports.

We collect data for several main outcomes measures. The CHART main outcome measure is 30-day self-reported smoking cessation at 6 months, and EQUIP has defined its own secondary outcome measure for 7-day, biochemically/proxy-verified smoking cessation at 6 months.

Five CHART sites are also conducting a sub-study of cotinine verified abstinence and are collecting biochemical verification using the same protocols in order to facilitate pooled analysis of verification data. The data collection process, cotinine analytic methods, and cutoffs are described in detail by Riley et al. in a separate article in this issue. Briefly, all patients who self-report being abstinent from tobacco for the 7 days preceding their 6-month survey, and who are not on nicotine replacement therapy, will be invited to provide a mailed salivary cotinine sample. They will be reimbursed $100 for the sample. Study staff follows an aggressive 30-day follow-up protocol involving multiple phone reminders, sample kit mailings, and an offer to meet the participant to collect the sample in their homes. Any participants who fail to provide samples within 30 days of their self-report are considered to be smokers.

In addition to biochemical verification via mailed salivary cotinine, EQUIP participants may also be considered abstinent under two conditions. First, participants who report they are abstinent may fail or refuse to provide a saliva sample but nominate a proxy to verify their smoking status. Staff will contact the proxy; if the proxy verifies tobacco abstinence, the smoker will be considered abstinent. Second, study participants who report tobacco abstinence but who are taking nicotine replacement therapy may provide an in-person expired-air carbon monoxide (CO) sample. If they provide a CO of <10 ppm, they will be considered abstinent.

We will assess cessation pharmacotherapy lifetime use and use in the past 30 days at baseline. To assess medication use any time during the study and in the past 30 days, we will follow the method of Williams et al. [21,22] and assess the type, dose, and number of days each medication was used.

### Study hypotheses (Table 4)

**Table 4:** Study hypotheses, measures, and analytic strategies

**Purpose**	**Variables**	**Analytic strategy**
**First aim: Main study outcomes**	Treatment condition and*:*	Logistic regression
Hypothesis 1	· Enrollment in quitline by 6 months	
Test the effects of warm handoff on quitline enrollment		
Hypothesis 2	Treatment condition and*:*	Logistic regression
Test the effects of warm handoff on post-discharge guideline-based counseling adherence	· Completing at least 3 quitline counseling sessions (binary)	
Hypothesis 3	Treatment condition and*:*	Logistic regression
Test the effects of warm handoff on 6-month abstinence outcomes	· 30-day abstinence	
	· 7-day abstinence	
	· Prolonged abstinence	
**Second Aim: Mediation analyses**	Treatment condition and 1-month*:*	Combined Poisson and logistic structural equation modeling
Hypothesis 4	· Satisfaction with care transition	
Effect of warm handoff on care transitions, enrollment, adherence, and pharmacotherapy utilization	· Enrollment in quitline	
	· No. of quitline sessions completed	
	· Pharmacotherapy use	
**Third aim: Costs and cost-effectiveness**	Treatment condition and:	-Average cost/arm
Hypothesis 5	· 30-day abstinence	-Incremental cost/quit
Warm handoff will be more costly, but also more cost-effective, than fax referrals	· Fixed/Variable costs	
	· Provider costs	-Providers
	· Participant costs	-Participants
		-Combined
**Feasibility, strengths/weaknesses of protocols**	Semistructured interviews	Qualitative analysis

We hypothesize that warm handoff will achieve higher quit rates because it will facilitate transitions in care. Warm handoff will increase the proportion of referred smokers who enroll in quitline services, compared to fax referral. Across both study arms, smokers who enroll in quitline services will receive level 4 intensity treatment (intensive inpatient treatment plus more than 1 month follow-up), and smokers who do not enroll will receive level 2 intensity treatment (intensive inpatient treatment but no follow-up, as defined in the recent *Cochrane Review of Interventions for Smoking Cessation in Hospitalized Patients)*[1]. Hence, quitline enrollment, adherence, and other evidence-based aspects of active tobacco treatment such as cessation medication use will be important mediators for study outcomes.

#### Costs for cost-effectiveness analyses

To assess intervention costs, for those randomized to the fax group, costs associated with completing and transmitting the fax form to the quitline will be assigned a standard time charge. For the warm handoff arm, counselors will track their time assisting with the three-way call to the quitline prospectively at the participant level. A standard per minute charge will be assigned to fax and telephone costs. Counselors at the quitline also will track their time prospectively as they interact with study participants by treatment arm. Personnel time will be valued at local wages plus benefits. In addition to program-borne costs, we will also add participants’ time costs consistent with their counselor time with costs valued at participants’ estimated hourly wages.

To assess short-term health care costs at 6 and 12 months, we will ask participants about their use of inpatient and outpatient health care resources, including what type of services (hospital stays, emergency department visits, general medicine and specialist physician visits) they used and how often. Although patients’ self-report of health care visits may either over or under report contact with providers, self-report is the least costly method of collecting data about health care use [23,24]. Since patients’ out-of-pocket expenses for these services will not accurately reflect the service value, we will use Medicare national fee structures to value the costs of these services.

We will estimate the costs of pharmacotherapy at the person level based upon retail prices documented prospectively through at least two on-line pharmacy websites, e.g., http://www.drugstore.com and http://www.walgreens.com. Finally, we will track participants’ lost work days in the first 6 months to assess indirect costs.

#### Hospital tobacco treatment staff satisfaction and recommendations for improvement

To assess the feasibility of the intervention for dissemination, we will use face-to-face semi-structured interviews to assess UKanQuit staff’s satisfaction with the study including fax and warm handoff referral of inpatients. Questions will cover overall satisfaction, strengths, weaknesses, and recommendations for improvement.

#### Treatment crossover

There is some potential for treatment crossover, in which patients in the fax referral arm begin to call the quitline from their hospital beds once they hear that patients in the warmhand off condition are contacting the quitline before discharge. In our experience, patients do not seem to communicate much with other patients—even patients sharing the same room. Because hospital stays are so short, only one in four patients smoke, and only half of our hospital beds are in two-patient rooms, it is not common to find two smokers in the same room for any length of time.

We are not sure how to reduce the likelihood that crossover occurs, but we will be able to monitor it. We will monitor the number of fax patients who contact the quitline prior to discharge by comparing patients’ date of discharge, which we will obtain from the electronic medical record, to the date of their enrollment in the quitline, which we will obtain from the quitline. Regardless of whether crossover occurs, all patients will be analyzed in the groups to which they were assigned, using an intent-to-treat analysis. However, if crossover occurs we will be able to describe the prevalence of these events and perform sensitivity analyses on the data by removing patients that crossed over and examining outcomes among the subset of patients that received the treatment to which they were assigned.

### Sample size justification and analyses

Our main outcome measure is 30-day point prevalence abstinence smoking cessation at 6 months post enrollment. We estimate our quit rate to be 9% in the control group and 15% in the treatment group, based on prior research [25,26]. We used formulas from Fleiss et al. (2003) [27] to calculate sample size. Assuming (1) a two-tailed alpha = 0.05 and power = 0.80; (2) intent-to-treat smoking cessation rates of 9% and 15% for fax referral and warm handoff, respectively; (3) a dichotomous outcome of biochemically verified 7-day abstinence at 12 months; a minimum of 994 participants are necessary to test primary effects. We also conducted power analyses for the proposed mediation models [28]. Analyses found sample sizes well short of 994 are sufficient for detecting mediation.

Table 4 outlines our study hypotheses and analyses. Data analyses include preliminary analyses to assess data quality and evaluate whether randomization achieved demographically equivalent study groups, outcome analyses, mediation analyses, qualitative analyses of staff and patient interviews, and cost analyses. Prior to initiating outcome analyses, we will examine frequency distributions for all variables, with particular attention to variable ranges, missing values, skewness and kurtosis (for continuous variables), and extreme unbalancedness in outcome proportions. Participants with missing outcome data will be retained in analyses but coded as smokers.

Our primary hypothesis (Hypothesis 3, Table 4) is that a significantly higher proportion of smokers receiving warm handoff will become enrolled in quitline services compared to smokers receiving fax referral. The primary analysis for Hypothesis 1 will be a comparison of 30-day point-prevalence abstinence at 6-month enrollment rates using logistic regression. The regression model will include a main effect term for group. An adjusted odds ratio for enrollment (and its confidence interval) will be computed. Covariates will be added as indicated by univariate analyses of baseline data. A number of other study hypotheses focus on other tobacco use outcomes, treatment utilization, and mediators of outcome. For analyses using structural equation modeling, missing data will be addressed either by employing full-information estimation algorithms or by first imputing missing data and treating input data as complete.

Our third aim is to examine the cost-effectiveness of warm handoff relative to fax referral**.** We anticipate that the warm handoff referral will be more costly and more effective than fax referral. The primary cost-effectiveness analysis will be set up as an incremental cost-effectiveness ratio (ICER). Incremental cost-effectiveness analysis identifies the marginal benefit of switching from one intervention to the other and is the ratio of the difference in costs divided by the difference in effectiveness between the two treatment options. The ICER will indicate the added cost per additional quitter for warm handoff versus fax referral, a metric that will allow comparisons to other smoking cessation economic studies. Given that all costs are short term (6–12 months), we will not discount either costs or benefits. Costs will be tracked from a societal perspective. The societal perspective sums all costs to all parties, including costs to the health care providers, to patients, and to third party payers. It is an estimate of the total cost to “society” for a treatment or intervention, as opposed to the costs for one party or one specific perspective, such as cost to the patient, or cost to the provider (physician offices).

We will also examine hospital staff satisfaction with intervention. Satisfaction survey items will be summarized using means and frequencies. Semistructured interviews will be analyzed using a qualitative software package. Transcribed interviews will be open-coded, identifying within the text key words, themes, and descriptions of behavior [29]. Subsequently, themes will be grouped into coding categories, and a code map will be developed that will allow us to categorize and retrieve core themes. Findings will also be used to strengthen future replications/adaptations of procedures.

### Data management

Research assistants will collect data on outcomes and costs, and will be responsible for entering data. The project director will conduct quality control on data collection and entry. The data manager will conduct initial data cleaning, identifying and tagging any crossovers, conversion into proper format for data analysis, and recoding using standard operating procedures.

## Discussion

This project is innovative as it potentially shifts current clinical practice by identifying sustainable methods for extending hospital care beyond discharge. The intervention we employ is novel as it capitalizes on the rise of quitlines as a universally accessible form of counseling care. It is highly responsive to recent changes in hospital care, in which primary care providers no longer attend their patients in the hospital but rely on inpatient “hospitalists” to provide care [30]. This has created a break in continuity of care that requires better communication and system linkages between in- and outpatient providers.

The project is significant because it addresses quality of hospital care for the most deadly preventable illness in the US; it addresses the under-researched area of post-discharge care; it describes rates of treatment uptake and adherence for a widely used but unevaluated practice (fax referral); it will evaluate the effectiveness and cost effectiveness of a novel approach for transferring care (warm handoff); and the findings of the trial will provide insight into how to more effectively address a systemic problem in the US health care system, namely, transitions in care. If effective, the intervention could be adapted for use in other types of interventions, for example, hospital staff could facilitate enrollment in text messages or web-based programs to inpatients’ mobile telephones prior to discharge to help patients prepare for cessation post-discharge.

The study has several limitations. It assesses the effects of a single course of treatment for tobacco dependence, however, many smokers may require multiple aided quit attempts to successfully quit. Our fidelity monitoring for intervention procedures involves having a supervisor observe treatment encounters, however, counselors will know they are being observed. Hence, the monitoring may fail to identify all inconsistencies in counseling procedures. Last, the intervention requires access to a free, proactive quitline that provides multiple treatment sessions for all smokers, regardless of insurance or income status. It will be poorly generalizable, however, if states continue to cut funding for and access to quitlines.

Hospitals and quitlines represent two excellent treatment systems that could, working together, potentially provide high-quality and low-cost treatment solutions for smokers. Identifying the best way to provide a seamless transition to outpatient care will make this potential a reality for the millions of smokers who enter US hospitals annually. Effect size differences between fax and warm handoff, although small, would have a significant public health impact because the prevalence of smoking in hospitals is high and the health effects of tobacco are so devastating. Multiplied across the millions of smokers admitted to hospitals each year, these improved outcomes could result in highly significant reductions in tobacco use and tobacco-related morbidity and mortality.

## Competing interests

The authors declare they have no competing interests.

## Authors’ contributions

KPR conceived of the study, led its design and coordination, and drafted the manuscript; BF conducted pilot studies to establish the feasibility of the trial; LMM revised study protocols, wrote sections of the manuscript, and directs implementation; EFE helped conceive the study and provided guidance on study design, measures, and analysis; BHC developed the quitline portion of the study; TIS is in charge of cost effectiveness and designed the measures and analyses of this portion of the trial; CLA helped design the quitline portion of the trial and will facilitate data collection of quitline enrollment and adherence; JJH assisted in developing the hospital counseling procedures and in manuscript development; KJP designed study analyses and measures; and DJC selected and coordinates collaboration with the secondary study site. All authors contributed to drafts of the manuscript and have read and approved the final manuscript.

## Funding

This project is funded by the National Heart, Lung, and Blood Institute (U01 HL105232-01). The content is solely the responsibility of the authors and does not necessarily represent the official views of the National Institutes of Health.
